# Prevalence and sociodemographic factors associated with vision difficulties in Ghana, Gambia, and Togo: a multi-country analysis of recent multiple Indicator cluster surveys

**DOI:** 10.1186/s12889-021-12193-7

**Published:** 2021-11-24

**Authors:** Abdul-Aziz Seidu, Pascal Agbadi, Precious Adade Duodu, Nutifafa Eugene Yaw Dey, Henry Ofori Duah, Bright Opoku Ahinkorah

**Affiliations:** 1 Centre for Gender and Advocacy, Takoradi Technical University, Takoradi, Ghana; 2 College of Public Health, Medical and Veterinary Sciences, James Cook University, Townsville, Queensland Australia; 3 Department of Nursing, College of Health Sciences, Kwame Nkrumah University of Science and Technology, Kumasi, Ghana; 4 Department of Nursing and Midwifery, School of Human and Health Sciences, University of Huddersfield, Queensgate, Huddersfield, England UK; 5 Department of Psychology, University of Ghana, P.O. Box LG, 84 Legon, Ghana; 6 Research Department, FOCOS Orthopaedic Hospital, Accra, Ghana; 7 School of Public Health, Faculty of Health, University of Technology Sydney, Sydney, Australia

**Keywords:** Vision difficulty, Prevalence, Sociodemographic factors, Ghana, Gambia, Togo, Multiple Indicator cluster surveys, Gender

## Abstract

**Background:**

The sense of sight is one of the important human sensory abilities that is required for independent functioning and survival. The highest burden of sight-related problems is recorded in low-and middle-income countries, especially in sub-Saharan Africa. Despite the burden, nationally representative analyses to understand the prevalence and determinants of vision difficulties are hard to find. Therefore, this study addressed this knowledge gap by estimating the prevalence of vision difficulties and its correlates in gender-stratified models in three West African countries: Ghana, Gambia, and Togo.

**Methods:**

The study used the most recent Multiple Indicator Cluster Surveys of Ghana (2017–2018), Gambia (2018), and Togo (2017). Summary statistics were used to describe the participants and logistic regression was used to perform the bivariate and multivariate analyses. The analyses were performed using Stata version 14 and the complex survey design of the datasets was accounted for using the ‘svyset’ command.

**Results:**

Gendered differences were observed for vision difficulties. More women than men reported vision difficulties in Ghana (men: 14.67% vs women: 23.45%) and Togo (men: 14.86% vs women: 23.61%), but more men than women reported vision difficulties in Gambia (men: 11.64% vs women: 9.76%). We also observed gender differences in how age, education, marital status, and region of residence were significantly associated with reported vision difficulties. The direction and magnitude of these relationships were different among men and women across the survey data in Ghana, Gambia, and Togo.

**Conclusion:**

The findings imply the need to tackle the existing gender inequities that are associated with vision difficulties to promote the quality of life of individuals, especially among older adults.

## Background

The global community has made significant efforts in reducing vision problems. In 1999, the World Health Organization (WHO) and the International Agency for the Prevention of Blindness initiated “Vision 2020: The Right to Sight” that aimed to eliminate avoidable blindness [1]. Also, the WHO adopted the 2014–2019 Global Action Plan dubbed ‘Universal Eye Health’ in 2013 [2]. This action plan was underpinned by principles and approaches such as universal access and equity, human rights, evidence-based practice, a life course approach, and empowerment of people with vision impairments (VIs) [2]. The action plan aimed to reduce the prevalence of avoidable VIs by 25% from 2010 to 2019 towards securing access to rehabilitation services for people with VIs [2, 3]. Nevertheless, VIs is a major global public health burden [4]. In this context, the term ‘vision impairment’ includes moderate and severe VIs as well as blindness [2]. According to the WHO, about one billion people globally have some level of VIs, mostly with near or distance vision [5]. Specifically, over 36 million people are blind and another 217 million live with moderate or severe VIs [6]. About 90% of the world’s visually impaired live in low-and middle-income countries (LMICs) [2], where the delivery of ophthalmic services is undermined by the scarcity of infrastructure, shortage of human resources and high user fees [6]. Nonetheless, about 80% of all VIs are largely preventable or treatable with minimal interventions [7]. VIs affect economic and educational opportunities, reduce quality of life, and increase the risk of death [3, 8].

As major causes of significant morbidity, VIs are largely attributable to refractive error, cataract, glaucoma, corneal opacities, diabetic retinopathy, trachoma, and presbyopia amongst others [3, 5]. The risk factors include diabetes mellitus, smoking, premature birth, rubella, and vitamin A deficiency [2]. With over 600 million people projected to be living with diabetes by 2040 [9], the number of people with diabetic retinopathy and resulting VIs is expected to rapidly rise, particularly among the economically active younger age groups in regions including sub-Saharan Africa (SSA) [1, 10, 11]. Age, gender (sex), nutritional factors among others are significant risk factors of VIs [12]. About 84% of people with VIs are aged 50 years and above as well as live in poor conditions. The female gender has been a significant predictor of VIs and a barrier to eye care services, with about two-thirds of all blind people globally being the poorest and oldest females. There is high prevalence and unfavourable odds of vision problems among women compared to their male counterparts [1, 13–17]. This required more concerted efforts to improve eye health, especially among females, thereby highlighting the global attention to dub the 2009 World Sight Day as ‘Gender and eye health: equal access to care’ [15]. With the rising sociodemographic status and life expectancy globally, there is a shift in the disease burden towards non-communicable diseases and disabilities including VIs [1]. Therefore, there was the need to include eye health in non-communicable and communicable disease frameworks to contribute to global initiatives that address ageing, marginalized and vulnerable groups [2]. This informed WHO’s adoption of the health system approach in its 2014–2019 Global Action Plan, which integrated eye care programmes into the wider health care system at all primary, secondary, and tertiary levels [2]. This sought to address the issue of access and cost for eye health care, as affected by social deprivation, using multisectoral preventive interventions [18]. In line with this, the Ghana’s National Health Insurance System [NHIS], for example, covered most ocular diseases, and almost every district has an ophthalmic nurse, as well as a very active private eye care sector [19, 20].

The sociodemographic, economic and contextual factors and the resultant socioeconomic position of individuals that influence vision functioning and the development of VIs underline the social determinants of health (SDOH) framework [21, 22]. The SDOH framework explains how structural differences in income, education, occupation, gender (sex), social class, and race/ethnicity amongst others influence health outcomes in populations [21, 22]. These structural mechanisms affect a wide range of health, functioning, and quality-of-life outcomes and invariably determine people’s exposure and vulnerability to health-compromising conditions [21]. In tandem with the 2014–2019 Global Action Plan, the SDOH framework conceptualize the health system itself within the context of SDOH and seek improved health systems and delivery to reduce VIs [21]. With about 90% of the world’s visually impaired living in LMICs such as Ghana, Gambia, and Togo [2], there is a need for the application of the SDOH framework and health equity approach [23]. To the best of our knowledge, there is a paucity of literature that used nationally representative data to explore the prevalence and predictors associated with vision difficulties in multiple countries in West Africa. Additionally, WHO’s 2014–2019 Global Action Plan stipulated the need for cutting-edge research in developing new and more cost-effective interventions, especially those that are applicable in LMICs [2]. By using the SODH framework, this study aimed to explore the gender disparity in the prevalence and sociodemographic factors associated with vision difficulty (a proxy for vision impairment) in persons aged 18–49 years living in Ghana, Gambia, and Togo by performing a multi-country analysis of recent Multiple Indicator Cluster Surveys (MICS).

## Methods

### Study design

This study is a secondary data analysis of nationally representative population-based cross-sectional survey data collected through the MICS. We specifically analyzed the most recent datasets of three countries in SSA [Ghana, 2017–2018; Gambia, 2018; and Togo, 2017]. The MICS was successfully conducted through a collaboration between the governmental statistical agencies of these countries and the United Nations International Children’s Emergency Fund (UNICEF) alongside other international donors. The MICS was designed purposely to aid participating countries in generating data for use in national development plans, policies, and programmes. Additionally, the MICS survey is expected to facilitate progress towards the Sustainable Development Goals (SDGs) and other internationally signed agreements. To this end, the MICS collects internationally comparable household survey data on a diverse range of indicators on children, men and women.

### Data collection procedure

The MICS collected nationally representative data using a multi-stage, stratified cluster sampling approach. Rural and urban areas representing the strata were initially identified across the administrative regions in all three countries. Within each of these strata, enumeration areas [EAs] were also identified and randomly selected as primary sampling units [PSUs]. The next process of the data collection included a listing and randomly sampling households within each of the selected EAs using systematic random sampling. Although, women of reproductive age [i.e., 15–49 years] and children were the target population of the survey, males of reproductive age were surveyed in half of the sampled households during the enumeration period. As a result, more females of reproductive age than males were sampled. The participant characteristics of Ghana, Gambia, and Togo are provided in Tables 1, 2 and 3, respectively.

**Table 1:** Socio-demographic characteristics of respondents (Ghana)

Variables	Males		Females	
	Frequency	Percentage	Frequency	Percentage
**Vision difficulty**				
No	**3676**	**85.53 [95% CI: 83.81, 86.72]**	**10,122**	**80.88 [95% CI: 79.88, 81.84]**
Yes	**632**	14.67 [**95% CI:** 13.27–16.19]	**2394**	19.12 [**95% CI:** 18.16–20.12]
**Age**				
< 20	516	12.0	1042	8.3
20–29	1463	34.0	4360	34.8
30–39	1250	29.0	4092	32.7
40+	1080	25.1	3023	24.2
**Educational level**				
Pre-primary or none	499	11.6	2660	21.3
Primary	445	10.3	2178	17.4
JSS/JHS/Middle	1647	38.2	4602	36.8
SSS/SHS/ Secondary	1220	28.3	2245	17.9
Higher	497	11.5	832	6.6
**Marital status**				
Currently married/in union	2371	55.0	8182	65.4
Formerly married/in union	195	4.5	1357	10.8
Never married/in union	1743	40.4	2978	23.8
**Health insurance**				
With insurance	1636	38.0	7044	56.3
Without insurance	2673	62.0	5473	43.7
**Wealth index**				
Poorest	741	17.2	2068	16.5
Poorer	659	15.3	2270	18.1
Middle	861	20.0	2514	20.1
Richer	970	22.5	2673	21.4
Richest	1078	25.0	2993	23.9
**Place of residence**				
Urban	2111	49.0	6373	50.9
Rural	2198	51.0	6144	49.1
**Region of residence**				
Western	441	10.2	1241	9.9
Central	349	8.1	1187	9.5
Accra	571	13.3	1697	13.6
Volta	337	7.8	953	7.6
Eastern	559	13.0	1502	12.0
Ashanti	1059	24.6	2978	23.8
Brong Ahafo	374	8.7	1149	9.2
Northern	386	9.0	1160	9.3
Upper West	131	3.0	362	2.9
Upper East	104	2.4	287	2.3

*JHS* Junior High School, *JSS* Junior Secondary School, *SHS* Senior High School, *SSS* Senior Secondary School

**Table 2:** Socio-demographic characteristics of respondents (Gambia)

Variables	Males		Females	
	Frequency	Percentage	Frequency	Percentage
**Vision difficulty**				
No	**3306**	**88.36 [95% CI: 86.94, 89.64]**	**10,615**	**90.25 [95% CI: 89.46, 90.98]**
Yes	**436**	**11.64 [95% CI: 10.36, 13.05]**	**1147**	**9.75 [95% CI: 9.02–10.54]**
**Age**				
< 20	405	10.8	1172	10.0
20–29	1566	41.9	4997	42.5
30–39	1076	28.8	3721	31.6
40+	695	18.6	1871	15.9
**Educational level**				
Pre-primary or none	1023	27.3	4773	40.6
Primary	532	14.2	1791	15.2
JSS/JHS/Middle	2188	58.5	5198	44.2
SSS/SHS/ Secondary	n/a	n/a	n/a	n/a
Higher	n/a	n/a	n/a	n/a
**Marital status**				
Currently married/in union	1707	45.6	8454	71.9
Formerly married/in union	48	1.3	721	6.1
Never married/in union	1987	53.1	2587	22.0
**Health insurance**				
With insurance	156	4.2	307	2.6
Without insurance	3586	95.8	11,455	97.4
**Wealth index**				
Poorest	527	14.1	2012	17.1
Poorer	593	15.9	2102	17.9
Middle	705	18.8	2252	19.1
Richer	884	23.6	2518	21.4
Richest	1032	27.6	2878	24.5
**Place of residence**				
Urban	2933	78.4	8432	71.7
Rural	809	21.6	3330	28.3
**Region of residence**				
Banjul	63	1.7	171	1.5
Kanifing	982	26.2	2756	23.4
Brikama	1661	44.4	4692	39.9
Mansakonko	121	3.2	436	3.7
Kerewan	302	8.1	1126	9.6
Kuntaur	113	3.0	483	4.1
Janjanbureh	205	5.5	703	6.0
Basse	295	7.9	1395	11.9

*na* Not applicable, *JHS* Junior High School, *JSS* Junior Secondary School, *SHS* Senior High School, *SSS* Senior Secondary School

**Table 3:** Socio-demographic characteristics of respondents (Togo)

Variables	Males		Females	
	Frequency	Percentage	Frequency	Percentage
**Vision difficulty**				
No	1664	85.14 [95% CI: 83.00, 87.05]	4896	76.39 [95% CI: 75.06, 77.66]
Yes	291	14.86 [95% CI: 12.95, 17.00]	1513	23.61 [95% CI: 22.34, 24.94]
**Age**				
< 20	187	9.57	520	8.11
20–29	712	36.43	2381	37.15
30–39	598	30.60	2070	32.29
40+	458	23.40	1439	22.45
**Educational level**				
Pre-primary or none	207	10.60	1977	30.84
Primary	480	24.56	2128	33.21
JSS/JHS/Middle	n/a	n/a	n/a	n/a
SSS/SHS/ Secondary	n/a	n/a	n/a	n/a
Secondary/Higher	1268	64.84	2304	35.95
**Marital status**				
Currently married/in union	1135	58.05	4752	74.14
Formerly married/in union	81	4.17	518	8.08
Never married/in union	739	37.78	1139	17.78
**Health insurance**				
With insurance	107	5.48	283	4.41
Without insurance	1848	94.52	6126	95.59
**Wealth index**				
Poorest	330	16.87	1045	16.31
Poorer	310	15.83	1144	17.85
Middle	398	20.37	1217	18.99
Richer	478	24.45	1455	22.70
Richest	439	22.48	1548	24.16
**Place of residence**				
Urban	929	47.52	3038	47.41
Rural	1026	52.48	3371	52.59
**Region of residence**				
Maritime	278	14.22	952	14.85
Plateaux	472	24.13	1438	22.44
Centrale	181	9.27	558	8.71
Kara	219	11.21	692	10.08
Savanes	214	10.97	774	12.07
Lome Commune	313	16.06	1030	16.07
Golfe Urbain	277	14.16	964	15.05

*na* Not applicable, *JHS* Junior High School, *JSS* Junior Secondary School, *SHS* Senior High School, *SSS* Senior Secondary School

### Measures

The datasets used in this study were collected by field enumerators using the Questionnaire for Individual Women and Men in the surveyed households of each country.

### Outcome variable

Seeing or vision difficulty was selected as the outcome variable for this study. This variable was measured using a single-item question [“Do you have difficulty seeing?”] with response options ranging from, “No difficulty”, “Some difficulty”, “A lot of difficulty” to “Cannot see at all”. This question which was adapted from the Washington Group Disability short set of questions on disability [WG-SS] and modified by the UNICEF has been shown to fairly classify adults with seeing disability [24, 25]. Although the adult version is yet to receive field testing, the child version of this question has been reported to possess sound psychometric properties [26]. For ease in interpretation, the responses to the variable were dichotomized by collapsing responses for “Some difficulty”, “A lot of difficulty” and “Cannot see at all” into “vision difficulty” and “No difficulty” into “no vision difficulty”. This recoding process followed a similar recoding and recommended cut-off points by previous literature [27, 28].

### Correlates

Correlates were selected based on variable availability and results from previous research [29, 30]. These correlates included age, gender (sex), educational level, marital status, health insurance coverage, household wealth index, rural-urban residence, and region of residence. The list of selected correlates for Ghana, Gambia, and Togo and their categorizations are reported in Tables 1, 2 and 3, respectively. Detailed descriptions of the questions measuring the variables are elsewhere [31]. Briefly, most of these variables were measured using straightforward questions and response formats (e.g., “Are you covered by any health insurance?” with “Yes” or “No” responses; “How old are you?” requiring participants to provide their actual age in numerals which was later categorized as seen on Table 1; “What is the highest level and grade or year of school you have attended?” with response ranging from “Pre-primary or none” to “Higher”). Other variables, on the other hand, were generated by computing the responses of several questions such as the construction of participants’ household wealth variable using household characteristics (e.g., internet access, number of rooms for sleeping and access to electricity, among others).

### Ethics and data availability

This is a secondary data analysis of publicly available data. Therefore no ethical approval was required for this study. The datasets used in this study is freely accessible at https://mics.unicef.org/surveys only after permission is sought from and granted by the UNICEF. In this study, permissions to use the datasets were sought and granted before accessing the data.

### Data preparation and analyses

Data analysis began with data cleaning and recoding, all done in Stata version 14 (Stata Corporation, College Station, TX, USA). Next, the data were weighted, allowing us to perform univariate analysis. As a result, frequencies and percentages were generated to describe the participants. Bivariate and multivariate analyses were subsequently performed using Chi-square and binary logistic regression, respectively. Before this, complex survey mode was declared using the ‘svyset’ command to enable the correction for clusters, stratification, and sample weights. This procedure is based on the recommendations of West, Sakshaug and Aurelien [32] who stressed the importance of accounting for possible analytical errors that are embedded within secondary datasets collected using complex sampling designs [32]. Once this correction was performed, bivariate analyses with Chi-square test were conducted to assess the relationship between the selected correlates with seeing difficulties, separately for males and females. Multivariate analyses with the “logistic” command were next conducted in two steps for both men and women datasets. First, correlates were individually regressed onto the outcome variable, and this is reported as the crude model in Tables 1 and 2. Second, all correlates were simultaneously regressed onto the outcome as reported in the adjusted model. We report both crude and adjusted odds ratios with their respective 95% confidence intervals (CI).

## Results

### Socio-demographic characteristics of respondents

#### Ghana

Table 1 shows the socio-demographic characteristics of the participants. It was found that the majority of the participants were aged 20–29 (34% males and 34.8% females). The majority also had JSS/JHS/middle school level of education (38.2% males and 36.8% females). With marital status, 55% of the males were currently married/in unions while 65.4% of the females were also currently married/in unions. In terms of health insurance subscription 38% of the males had subscribed whilst 56.3% of the females also subscribed. Almost a quarter of the respondents were from Ashanti region (24.6% males and 23.8% females).

#### Gambia

It was found that the majority of the participants were aged 20–29 (41.9% males and 42.5% females). The majority also had JSS/JHS/middle school level of education (58.5% males and 44.2% females). With marital status, 45.6% of the males were currently married/in unions while 71.9% of the females were also currently married/in unions. In terms of health insurance subscription the majority (95.8% males and 97.4% females) have not subscribed to health insurance. The majority were in urban areas (78.4% males and 71.7% females) (Table 2).

#### Togo

The socio-demographic characteristics of the participants are showed in Table 3. It was found that the majority of the participants were aged 20–29 (36.4% Males and 37.2% females). The majority also had secondary/higher level of education (64.8% males and 35.9% females). With marital status, 58.0% of the males were currently married/in unions while 74.1% of the females were also currently married/in unions. In terms of health insurance subscription the majority (94.5% males and 95.6% females) have not subscribed to health insurance. More than half (52.5% males and 52.6% females) were in rural areas.

### Ghana: summary statistics of study variables, sociodemographic variables regressed on vision difficulty, stratified by gender

The proportion of women reporting some form of vision difficulty was 19.10% and that of men was 14.67% (Table 4). The distribution of vision difficulty proportions across sociodemographic characteristics of the respondents was unequal (Table 4). In the adjusted model, gendered differences were observed in the relationship between sociodemographic factors and vision difficulty (Table 4). Age was positively associated with reported vision difficulties among both men and women, but the odds of reporting vision difficulty was higher among men (Table 4). Although education was not a significant correlate of vision difficulties among men, it was significant among women; compared to women with post-secondary education, women with secondary or primary education were more likely to report some form of vision difficulty (Table 4). Household wealth was significant among men but not among women. Specifically, compared to the richest wealth quintile, men of the middle household wealth index were more likely to report vision difficulties (Table 4). Although the region of residence was a significantly associated factor among both men and women, the direction and magnitude of the odds ratios were different (Table 4). Compared to the Upper West region, men who resided in the remaining nine regions except the Western Region had a higher likelihood of reporting vision difficulties (Table 4). Among women, we observed negative and positive relationships with reporting vision difficulties in Upper West and Greater Accra, respectively (Table 4).

**Table 4:** Ghana: summary statistics of study variables, sociodemographic variables regressed on vision difficulty, stratified by gender

Variables (Ghana)	Males	Females	Males		Females	
	*N* = 4309	*N* = 12,517	*N* = 4308		*N* = 12,516	
Prevalence	14.67[**95% CI:** 13.27–16.19]	19.12[**95% CI:** 18.16–20.12]	Crude odds ratio	Adjusted odds ratio	Crude odds ratio	Adjusted odds ratio
**Age**	***P*** **< 0.001**	***P*** **< 0.001**				
< 20	11.75	18.19	Ref	Ref	Ref	Ref
20–29	12.36	14.30	1.059 [0.692,1.619]	1.122 [0.714,1.763]	0.750* [0.588,0.957]	0.803 [0.627,1.029]
30–39	10.53	15.35	0.884 [0.575,1.359]	1.074 [0.627,1.840]	0.816 [0.647,1.029]	0.929 [0.713,1.212]
40+	24.00	31.51	2.371*** [1.556,3.612]	3.109*** [1.739,5.561]	2.069*** [1.610,2.659]	2.400*** [1.786,3.225]
**Educational level**	***P*** **= 0.145**	***P*** **= 0.019**				
Pre-primary or none	17.81	18.91	1.170 [0.754,1.815]	0.790 [0.468,1.335]	1.455* [1.074,1.970]	1.233 [0.877,1.734]
Primary	16.64	20.25	1.078 [0.667,1.743]	0.834 [0.471,1.475]	1.584** [1.152,2.180]	1.328 [0.944,1.867]
JSS/JHS/Middle	14.35	19.59	0.905 [0.627,1.305]	0.655 [0.423,1.013]	1.520** [1.136,2.033]	1.365* [1.005,1.853]
SSS/SHS/ Secondary	12.72	19.3	0.787 [0.535,1.158]	0.706 [0.460,1.084]	1.492** [1.115,1.996]	1.509** [1.126,2.023]
Higher	15.62	13.82	Ref	Ref	Ref	Ref
**Marital status**	***P*** **= 0.001**	***P*** **< 0.001**				
Currently married/in union	15.65	19.46	1.276* [1.016,1.604]	0.765 [0.523,1.120]	1.185* [1.034,1.358]	0.903 [0.749,1.088]
Formerly married/in union	20.61	21.92	1.786* [1.045,3.052]	1.130 [0.617,2.069]	1.377** [1.108,1.712]	0.892 [0.689,1.156]
Never married/in union	12.69	16.93	Ref	Ref	Ref	Ref
**Health insurance**	***P*** **= 0.327**	***P*** **= 0.196**				
With insurance	15.06	18.6	Ref	Ref	Ref	Ref
Without insurance	14.44	19.8	0.952 [0.732,1.238]	0.847 [0.629,1.141]	1.081 [0.946,1.235]	1.022 [0.884,1.182]
**Wealth index**	***P*** **= 0.125**	***P*** **= 0.002**				
Poorest	16.25	19.26	1.324* [1.032,1.697]	1.648 [0.991,2.741]	0.817** [0.716,0.932]	1.085 [0.819,1.435]
Poorer	15.45	17.77	1.339* [1.014,1.770]	1.456 [0.867,2.445]	0.785** [0.679,0.908]	0.881 [0.675,1.150]
Middle	16.17	18.15	1.329* [1.011,1.747]	1.606* [1.040,2.481]	0.823** [0.716,0.947]	0.904 [0.715,1.144]
Richer	14.21	19.63	1.142 [0.869,1.502]	1.335 [0.890,2.002]	0.944 [0.825,1.080]	0.981 [0.804,1.198]
Richest	12.34	20.43	Ref	Ref	Ref	Ref
**Place of residence**	***P*** **= 0.242**	***P*** **= 0.044**				
Urban	13.91	19.08	Ref	Ref	Ref	Ref
Rural	15.41	19.17	1.127 [0.843,1.506]	0.996 [0.683,1.450]	1.005 [0.875,1.155]	1.150 [0.958,1.381]
**Region of residence**	***P*** **< 0.001**	***P*** **< 0.001**				
Western	6.21	19.5	0.949 [0.394,2.283]	1.207 [0.487,2.992]	1.052 [0.744,1.487]	1.127 [0.770,1.648]
Central	19.08	20.02	3.376*** [1.831,6.226]	4.931*** [2.483,9.792]	1.087 [0.786,1.504]	1.137 [0.797,1.623]
Accra	12.54	24.46	2.053* [1.119,3.767]	2.875** [1.437,5.751]	1.407* [1.062,1.864]	1.550** [1.121,2.144]
Volta	20.17	21.62	3.619*** [2.003,6.538]	4.530*** [2.412,8.509]	1.198 [0.879,1.633]	1.230 [0.883,1.712]
Eastern	17.57	17.22	3.053*** [1.649,5.652]	3.909*** [1.996,7.654]	0.903 [0.634,1.288]	0.906 [0.615,1.335]
Ashanti	15.05	19.45	2.537** [1.313,4.902]	3.252*** [1.663,6.359]	1.049 [0.780,1.409]	1.120 [0.813,1.543]
Brong Ahafo	17.18	16.18	2.970***[1.601,5.510]	3.588*** [1.860,6.921]	0.839 [0.604,1.165]	0.877 [0.614,1.251]
Northern	14.74	14.48	2.475** [1.267,4.835]	2.615** [1.317,5.192]	0.735 [0.526,1.027]	0.761 [0.538,1.078]
Upper West	11.28	11.59	1.821 [0.958,3.465]	2.033* [1.041,3.972]	0.570** [0.398,0.814]	0.570** [0.397,0.820]
Upper East	6.53	18.71	Ref	Ref	Ref	Ref

Exponentiated coefficients; 95% confidence intervals in brackets; significant adjusted values are boldened; *JHS* Junior High School, *JSS* Junior Secondary School, *SHS* Senior High School, *SSS* Senior Secondary Schooll

* *p* < 0.05, ** *p* < 0.01, *** *p* < 0.001

### Gambia: summary statistics of study variables, sociodemographic variables regressed on vision difficulty, stratified by gender

The proportion of Gambian men reporting some form of vision difficulty was 11.64% and that of Gambian women was 9.76% (Table 5). The distribution of vision difficulty proportions across socio-demographic characteristics of the respondents was unequal (Table 5). In the adjusted model, gendered differences were seen in the association between socio-demographic factors and vision difficulties (Table 5). Compared to men and women less than 20 years old, men and women who were 40 years and above were more likely to report vision difficulties in the Gambia. Education level was a significant correlate of vision difficulties among Gambian women only. Specifically, compared with women with middle school education level, women with pre-primary or no formal education have a lower likelihood of reporting vision difficulties. Marital status was not a significant correlate among men, but it was significant in the women model. Specifically, compared to unmarried women, women who were currently married/in the union were less likely to report vision difficulties. Region of residence was associated with reports of vision difficulties among both men and women in The Gambia. Among men, residing in Mansakonko or Janjanbureh was negatively associated with reported vision difficulties relative to residents of Basse. Among Gambian women, residing in Kanifing or Brikama or Kerewan was positively associated with reporting vision difficulties.

**Table 5:** Gambia: summary statistics of study variables, sociodemographic variables regressed on vision difficulty, stratified by gender

Variables (Gambia)	Males	Females	Males		Females	
	*N* = 3742	*N* = 11,762	*N* = 3742		*N* = 11,762	
**Prevalence**	**11.64 [95% CI: 10.36, 13.05]**	**9.75[95% CI: 9.02–10.54]**	**Crude odds ratio**	**Adjusted odds ratio**	**Crude odds ratio**	**Adjusted odds ratio**
**Age**	***P*** **< 0.001**	***P*** **< 0.001**				
< 20	10.43	10.17	Ref	Ref	Ref	Ref
20–29	11.06	7.32	1.069 [0.707,1.615]	1.122 [0.740,1.700]	0.698* [0.502,0.969]	0.809 [0.560,1.169]
30–39	8.27	9.25	0.775 [0.473,1.269]	0.945 [0.532,1.679]	0.900 [0.648,1.250]	1.309 [0.875,1.959]
40+	8.27	16.98	1.997** [1.260,3.165]	2.601** [1.415,4.780]	1.806*** [1.298,2.513]	2.814*** [1.807,4.383]
**Educational level**	***P*** **= 0.084**	***P*** **< 0.001**				
Pre-primary or none	10.94	7.77	0.916 [0.670,1.254]	0.882 [0.621,1.252]	0.636*** [0.525,0.770]	0.640*** [0.500,0.820]
Primary	12.25	9.35	1.041 [0.708,1.531]	0.957 [0.637,1.438]	0.778 [0.595,1.017]	0.830 [0.625,1.103]
JSS/JHS/Middle	11.81	11.71	Ref	Ref	Ref	Ref
SSS/SHS/ Secondary	n/a	n/a	n/a	n/a	n/a	n/a
Higher	n/a	n/a	n/a	n/a	n/a	n/a
**Marital status**	***P*** **= 0.359**	***P*** **< 0.001**				
Currently married/in union	12.04	8.83	1.087 [0.832,1.420]	0.810 [0.531,1.235]	0.742** [0.600,0.917]	0.682* [0.507,0.917]
Formerly married/in union	16.45	14.14	1.564 [0.400,6.113]	1.167 [0.280,4.864]	1.261 [0.895,1.778]	0.936 [0.629,1.395]
Never married/in union	11.18	11.55	Ref	Ref	Ref	Ref
**Health insurance**	***P*** **= 0.676**	***P*** **< 0.001**				
With insurance	7.18	16.01	Ref	Ref	Ref	Ref
Without insurance	11.84	9.58	1.735 [0.745,4.039]	2.141 [0.939,4.881]	0.556* 0.312,0.990]	0.822 [0.439,1.538]
**Wealth index**	***P*** **= 0.322**	***P*** **< 0.001**				
Poorest	10.99	8.15	0.743 [0.549,1.005]	0.980 [0.534,1.799]	0.456*** [0.376,0.553]	1.003 [0.669,1.503]
Poorer	11.74	8.13	0.892 [0.653,1.218]	0.997 [0.593,1.677]	0.546*** [0.446,0.669]	0.844 [0.596,1.196]
Middle	10.12	8.48	0.774 [0.553,1.083]	0.820 [0.515,1.305]	0.541*** [0.437,0.670]	0.796 [0.565,1.121]
Richer	13	9.39	0.910 [0.669,1.238]	1.068 [0.709,1.608]	0.678*** [0.551,0.835]	0.757 [0.549,1.044]
Richest	11.79	13.37	Ref	Ref	Ref	Ref
**Place of residence**	***P*** **= 0.031**	***P*** **< 0.001**				
Urban	11.92	11.03	Ref	Ref	Ref	Ref
Rural	10.63	6.52	0.879 [0.670,1.154]	1.146 [0.669,1.964]	0.562*** [0.461,0.686]	0.965 [0.699,1.332]
**Region of residence**	***P*** **= 0.014**	***P*** **< 0.001**				
Banjul	12.5	10.12	0.913 [0.555,1.503]	0.866 [0.475,1.577]	2.307*** [1.548,3.437]	1.481 [0.923,2.375]
Kanifing	12.19	11.24	0.886 [0.537,1.462]	0.912 [0.504,1.650]	2.595*** [1.833,3.673]	1.772** [1.168,2.690]
Brikama	12.07	12.06	0.876 [0.557,1.377]	0.905 [0.530,1.547]	2.809*** [1.992,3.962]	2.137*** [1.495,3.053]
Mansakonko	7.82	5.48	0.541* [0.317,0.926]	0.515* [0.290,0.916]	1.187 [0.823,1.713]	0.970 [0.669,1.405]
Kerewan	10.57	9.05	0.754 [0.439,1.296]	0.731 [0.413,1.293]	2.040*** [1.391,2.991]	1.788** [1.223,2.615]
Kuntaur	11.25	4.81	0.809 [0.488,1.341]	0.795 [0.459,1.376]	1.036 [0.700,1.532]	0.915 [0.594,1.407]
Janjanbureh	6.62	5.71	0.453** [0.278,0.738]	0.432** [0.257,0.726]	1.240 [0.796,1.933]	1.074 [0.691,1.669]
Basse	13.55	4.65	Ref	Ref	Ref	Ref

Exponentiated coefficients; 95% confidence intervals in brackets; significant adjusted values are boldened; *JJHS* Junior High School, *JSS* Junior Secondary School, *SHS* Senior High School, *SSS* Senior Secondary School,

*na* Not applicable

* *p* < 0.05, ** *p* < 0.01, *** *p* < 0.001

### Togo: summary statistics of study variables, sociodemographic variables regressed on vision difficulty, stratified by gender

Nineteen percent of women (19.12%) and 14.86% of men reported vision difficulties. The proportion of men or women reporting vision difficulties also varies across the socio-demographic divide in Togo. Age and education level were not significant in the men model, but they were significant among women in Togo. Compared to women less than 20 years, women who were 30–39 years or 40 years and above have a higher likelihood of reporting vision difficulties. Compared to women with secondary and higher education, women with pre-primary or no formal education have a lower likelihood of reporting vision difficulties. Marital status was significant in both men and women but with differences in the direction and magnitude of the association. Among men, having formerly married was positively associated with reporting vision difficulties. Among women, women who were currently married/in union were less likely to report vision difficulties compared with unmarried women. Also, the region of residence was significant in both men and women models. Among men, residing in Maritime, Plateaux, Kara, Lome Commune, and Golfe Urbain were positively associated with reported vision difficulties relative to residents of Centrale. Among women, residents of Maritime and Golfe Urbain were more likely to report vision difficulties relative to residents of Centrale (Table 6).

**Table 6:** Togo: summary statistics of study variables, sociodemographic variables regressed on vision difficulty, stratified by gender

Variables (TOGO)	Males	Females	Males		Females	
	*n* = 1955	*n* = e6409	*n* = 1955		*n* = 6409	
**Prevalence**	**14.86 [95% CI: 12.95, 17.00]**	**23.61 [95% CI: 22.34, 24.94]**	**Crude odds ratio**	**Adjusted odds ratio**	**Crude odds ratio**	**Adjusted odds ratio**
**Age**	***P*** **< 0.001**	***P*** **< 0.001**				
< 20	12.86	18.98	Ref.	Ref.	Ref.	Ref.
20–29	10.01	16.09	0.753 [0.429,1.322]	0.737 [0.384,1.417]	0.818 [0.593,1.129]	1.003 [0.722,1.392]
30–39	11.66	22.23	0.894 [0.495,1.618]	0.787 [0.376,1.647]	1.220 [0.850,1.750]	1.677** [1.172,2.400]
40+	27.41	39.73	2.558** [1.350,4.846]	2.325 [0.962,5.620]	2.814*** [2.001,3.957]	4.029*** [2.840,5.715]
**Education level**	***P*** **< 0.05**	***P*** **< 0.001**				
Pre-primary or none	12.75	21.36	0.883 [0.476,1.638]	0.767 [0.402,1.464]	0.895 [0.724,1.108]	0.756* [0.591,0.967]
Primary	17.49	26.08	1.280 [0.907,1.808]	1.001 [0.646,1.550]	1.163 [0.991,1.366]	0.994 [0.827,1.194]
JSS/JHS/Middle	n/a	n/a	n/a	n/a	n/a	n/a
SSS/SHS/ Secondary	n/a	n/a	n/a	n/a	n/a	n/a
Secondary and Higher	14.21	23.27	Ref.	Ref.	Ref.	Ref.
**Marital status**	***P*** **< 0.001**	***P*** **< 0.001**				
Currently married/in union	16.66	22.33	1.675* [1.124,2.496]	1.195 [0.695,2.053]	0.950 [0.762,1.184]	0.683*** [0.546,0.853]
Formerly married/in union	27.86	36.22	3.237*** [1.648,6.357]	2.463* [1.111,5.462]	1.876*** [1.335,2.637]	1.055 [0.740,1.505]
Never married/in union	10.66	23.24	Ref.	Ref.	Ref.	Ref.
**Health insurance**	***P*** **= 0.421**	***P*** **= 0.088**				
With insurance	12.3	28.58	Ref.	Ref.	Ref.	Ref.
Without insurance	15.01	23.38	1.259 [0.634,2.500]	1.642 [0.829,3.251]	0.763 [0.562,1.036]	1.015 [0.730,1.412]
**Wealth index**	***p*** **= 0.141**	***P*** **< 0.001**				
Poorest	11.02	18.13	0.719 [0.414,1.250]	0.605 [0.278,1.317]	0.618*** [0.468,0.818]	0.941 [0.636,1.390]
Poorer	19.65	20.48	1.421 [0.902,2.237]	1.307 [0.652,2.620]	0.719* [0.552,0.937]	0.969 [0.677,1.387]
Middle	17.69	24.49	1.249 [0.802,1.944]	1.131 [0.647,1.978]	0.906 [0.732,1.120]	1.030 [0.794,1.337]
Richer	12.2	26.34	0.807 [0.512,1.273]	0.833 [0.516,1.346]	0.999 [0.801,1.246]	1.111 [0.883,1.397]
Richest	14.69	26.37	Ref.	Ref.	Ref.	Ref.
**Place of residence**	***p*** **= 0.670**	***P*** **< 0.001**				
Urban	13.05	26.67	Ref.	Ref.	Ref.	Ref.
Rural	16.5	20.86	1.316 [0.950,1.824]	1.258 [0.707,2.239]	0.725** [0.594,0.884]	0.914 [0.605,1.381]
**Region of residence**	***P*** **< 0.001**	***P*** **< 0.001**				
Maritime	24.88	27.59	5.961*** [2.827,12.57]	5.634*** [2.663,11.92]	1.569** [1.165,2.113]	1.448* [1.055,1.987]
Plateaux	19.13	21.34	4.259*** [2.058,8.816]	4.481*** [2.153,9.322]	1.117 [0.762,1.637]	1.023 [0.693,1.510]
Centrale	5.26	19.54	Ref.	Ref.	Ref.	Ref.
Kara	13.78	23.95	2.812** [1.391,5.683]	3.178** [1.558,6.482]	1.297 [0.968,1.737]	1.323 [0.998,1.755]
Savanes	5.78	13.72	1.104 [0.488,2.500]	**1.238 [0.539,2.843]**	0.655* [0.444,0.966]	0.761 [0.509,1.138]
Lome Commune	12.91	27.78	2.667* [1.219,5.838]	3.336** [1.361,8.181]	1.584*** [1.238,2.028]	1.269 [0.885,1.822]
Golfe Urbain	14.12	28.67	2.959** [1.420,6.167]	3.520** [1.522,8.143]	1.655*** [1.292,2.121]	1.438* [1.021,2.026]

Exponentiated coefficients; 95% confidence intervals in brackets; significant adjusted values are boldened; *JHS* Junior High School, *JSS* Junior Secondary School, *SHS* Senior High School, *SSS* Senior Secondary School,

*na* Not applicable

* *p* < 0.05, ** *p* < 0.01, *** *p* < 0.001

## Discussion

The sense of sight is one of the important human functions given that is required for independent functioning and survival. We report on the gender disparities in the prevalence and sociodemographic predictors of vision difficulties in three countries in West Africa namely Ghana, Gambia, and Togo. The prevalence of vision difficulties in males was 14.67%, 11.64% and 14.86% for Ghana, Gambia, and Togo, respectively. The prevalence of vision difficulties among females was 19.12%, 9.76%, and 23.61%, respectively. This highlights variations in the burden of vision difficulties in both males and females across the three countries under review, with females in Ghana and Togo recording higher prevalence. On the contrary, the burden of vision difficulties was lower in the female participants of The Gambia relative to the burden in the males. Gender inequalities in vision difficulties to the disadvantage of women have been reported in the literature [14]. These gender differences have been attributed to factors such as gender inequalities in access to health care as well as infrequent and ineffective use of health care services due to gender socialization [13, 14].

Consistently, people aged 40 years and above in all the three countries had a significantly higher likelihood of having vision difficulties as compared to their counterparts below 20 years. This finding was consistent in all the female participants in Ghana, Gambia and Togo as well as all the male participants in Ghana and Gambia except Togo. Literature suggests that as people age, the anatomy and physiology of the eye changes [33]. This is likely to be the case for the observations we made in this present study. Although, the cause of vision loss is multifactorial and cannot be attributable to ageing alone, it appears that ageing is a leading factor. This finding is consistent with previous studies that reported a higher likelihood of vision difficulties with ageing [34–36]. Age-related changes in the anatomy and physiology of the eye, therefore, largely explain the observation that people aged 40 years and above had a higher likelihood of vision difficulties.

Educational level was found to be a significant predictor of vision difficulties in only Ghana and Gambia among the female sub-samples. In Ghana, it was observed that relative to people with higher education, those with Junior and Senior Secondary School (JSS and SSS) education had higher likelihoods of having vision difficulties. The result is consistent with a previous study that also reported that relative to those with a university education, individuals with lower educational attainments had a higher likelihood of vision difficulties [34]. Another systematic review reported that educational attainment was inversely associated with vision difficulties [37]. Higher educational attainment provides greater knowledge and access to healthcare resources and practices which are important for improved health including better eyesight [38, 39]. Participants with lower educational level may therefore not be benefitting from these resources. However, it was observed in The Gambia that relative to females with Junior High School education, those with Primary or no-education had decreased likelihood of vision difficulties. Nevertheless, it is difficult to be conclusive about the direction of the observed association as vision difficulty itself can also affect academic attainment [40].

Household wealth status was found to be significantly associated with vision difficulties among only the male participants in Ghana. We found that relative to males from households in the richest wealth quantile, their counterparts from middle wealth quintiles were associated with higher odds of vision difficulties. Our explanation for this observation may be related to the fact that individuals from the richest households are perhaps able to afford the cost of early diagnosis and treatment for eye conditions which results in a low burden of vision difficulties. Our findings corroborate the results of other reviews which also reported that income status was inversely associated with vision difficulties [37]. Governments of these three countries should develop pro-poor policies to help improve the financial status of their populations and its associated benefits on health including vision care.

Our findings also revealed a decreased likelihood of vision difficulties among Gambian and Togolese females who were currently in marriage compared to their counterparts who were never married. On the contrary, we found that Togolese males who were formerly married had a higher likelihood of having vision difficulties compared to those who were never married. Zheng et al. [41] reported that having unmarried status is associated with an increased risk of vision difficulty [41]. One plausible explanation is that marital status provides economic and emotional support for early treatment of vision difficulties. Moreover, the lack of support for self-care and social isolation may partly explain the high burden of vision difficulties among those with unmarried status, especially in the elderly populations [41, 42]. Therefore, couple-friendly services may be considered in increasing the uptake of vision care services.

The study also revealed some regional and sub-national variations in the burden of vision difficulties across Ghana, Gambia, and Togo in both the male and female participants. This calls for further research to explain the contextual factors explaining the intra-regional variations in the burden of visual difficulties. From a policy standpoint, our findings highlight substantial burdens of vision difficulties in Ghana, Gambia, and Togo. There is a need for health interventions that address the vision care needs of older individuals. Our findings also highlight the need to support individuals who were formerly in marriage, especially among older males to encourage the early treatment of vision difficulties. The observed gendered differences in how age, education level, marital status, and region of residence associate with reported vision difficulties also underscores the need to address the existing gender inequities that affect vision difficulties and other health outcomes.

### Strengths and limitations

The strengths in our paper lie in our use of nationally representative data from three countries in SSA to investigate the correlates of vision difficulties. We unearthed the gender disparities in the burden and correlates of vision difficulties. Nevertheless, given the use of cross-sectional design, the associations observed in our paper do not infer causal relationships. Moreover, the assessments of vision difficulties were subjective and was based on respondents’ views of visual acuity instead of objective medical evaluations. Therefore, it is worth mentioning that there is a potential risk for misclassification bias in the assessment of vision difficulties.

## Conclusions

The study revealed gender differences in the burden of vision difficulties in Ghana, Gambia, and Togo. There were also observable gender differences in how age, educational level, marital status, and region of residence were associated with reported vision difficulties. The findings imply the need to tackle the existing gender inequities that affect visual difficulties to promote the quality of life of individuals, especially the elderly.

## Data Availability

The datasets used in this study is freely accessible at https://mics.unicef.org/surveys only after permission is sought from and granted by the UNICEF.

## Abbreviations

VIs: Visual Impairments
SDH: Social Determinants of Health
LMICs: Low-and Middle-Income Countries
SSA: Sub-Saharan Africa
AOR: Adjusted Odds Ratio
CI: Confidence Interval
DHS: Demographic and Health Survey
EA: Enumeration Areas
MICS: Multiple Indicator Cluster Survey
PSU: Primary Sampling Units
SDG: Sustainable Development Goals
UNICEF: United Nations International Children’s Emergency Fund
WHO: World Health Organization
JHS: Junior High School
JSS: Junior Secondary School
SHS: Senior High School
SSS: Senior Secondary School

## References

[1] Adelson JD, Bourne RRA, Briant PS, Flaxman SR, Taylor HRB, Jonas JB (2021). Causes of blindness and vision impairment in 2020 and trends over 30 years, and prevalence of avoidable blindness in relation to VISION 2020: the right to sight: an analysis for the global burden of disease study. Lancet Glob Health.

[2] World Health Organization. WHO | Universal eye health: a global action plan 2014–2019. WHO. Geneva: World Health Organization; 2013. Available from: http://www.who.int/blindness/actionplan/en/.

[3] Bourne RRA, Flaxman SR, Braithwaite T, Cicinelli MV, Das A, Jonas JB, et al. Magnitude, temporal trends, and projections of the global prevalence of blindness and distance and near vision impairment: a systematic review and meta-analysis. Lancet Glob Health. 2017;5(9):e888–97. 10.1016/S2214-109X(17)30293-0. 10.1016/S2214-109X(17)30293-0 28779882

[4] Keeffe J, Resnikoff S. Prevalence and causes of vision impairment and blindness: the global burden of disease. In: Khanna RC, Rao GN, Marmamula S, editors. Innovative approaches in the delivery of primary and secondary eye care. Cham: Springer International Publishing; 2019. p. 7–20. (essentials in ophthalmology). Available from: 10.1007/978-3-319-98014-0_2.

[5] World Health Organization. Vision impairment and blindness. Geneva: 2020. Available from: https://www.who.int/news-room/fact-sheets/detail/blindness-and-visual-impairment.

[6] Bechange S, Jolley E, Virendrakumar B, et al. Strengths and weaknesses of eye care services in sub-Saharan Africa: a meta-synthesis of eye health system assessments. BMC Health Serv Res. 2020;20:381:1–8. 10.1186/s12913-020-05279-2. 10.1186/s12913-020-05279-2 PMC7203845 32375761

[7] Stevens GA, White RA, Flaxman SR, Price H, Jonas JB, Keeffe J, et al. Global prevalence of vision impairment and blindness: magnitude and temporal trends, 1990–2010. Ophthalmology. 2013;120(12):2377–84. 10.1016/j.ophtha.2013.05.025. 10.1016/j.ophtha.2013.05.025 23850093

[8] Eckert KA, Carter MJ, Lansingh VC, Wilson DA, Furtado JM, Frick KD, Resnikoff S (2015). A simple method for estimating the economic cost of productivity loss due to blindness and moderate to severe visual impairment. Ophthalmic Epidemiol.

[9] Ogurtsova K, da Rocha Fernandes JD, Huang Y, Linnenkamp U, Guariguata L, Cho NH, et al. IDF diabetes atlas: global estimates for the prevalence of diabetes for 2015 and 2040. Diabetes Res Clin Pract. 2017;128:40–50. 10.1016/j.diabres.2017.03.024. 10.1016/j.diabres.2017.03.024 28437734

[10] Leasher JL, Bourne RRA, Flaxman SR, Jonas JB, Keeffe J, Naidoo K, et al. Global estimates on the number of people blind or visually impaired by diabetic retinopathy: a Meta-analysis from 1990 to 2010. Diabetes Care. 2016;39(9):1643–9. 10.2337/dc15-2171. 10.2337/dc15-2171 27555623

[11] Yau JWY, Rogers SL, Kawasaki R, Lamoureux EL, Kowalski JW, Bek T, et al. Global prevalence and major risk factors of diabetic retinopathy. Diabetes Care. 2012;35(3):556–64. 10.2337/dc11-1909. 10.2337/dc11-1909 PMC3322721 22301125

[12] Nuertey BD, Amissah-Arthur KN, Addai J, Adongo V, Nuertey AD, Kabutey C (2019). Prevalence, Causes, and Factors Associated with Visual Impairment and Blindness among Registered Pensioners in Ghana. J Ophthalmol.

[13] Prasad M, Malhotra S, Kalaivani M, Vashist P, Gupta SK (2020). Gender differences in blindness, cataract blindness and cataract surgical coverage in India: a systematic review and meta-analysis. Br J Ophthalmol.

[14] Rius Ulldemolins A, Benach J, Guisasola L, Artazcoz L (2019). Why are there gender inequalities in visual impairment?. Eur J Pub Health.

[15] Ye Q, Chen Y, Yan W, Wang W, Zhong J, Tang C (2020). Female Gender Remains a Significant Barrier to Access Cataract Surgery in South Asia: A Systematic Review and Meta-Analysis. J Ophthalmol.

[16] Mousa A, Courtright P, Kazanjian A, Bassett K (2014). Prevalence of visual impairment and blindness in upper Egypt: a gender-based perspective. Ophthalmic Epidemiol.

[17] Doyal L, Das-Bhaumik RG (2018). Sex, gender and blindness: a new framework for equity. BMJ Open Ophthalmol.

[18] Bezabih L, Abebe TW, Fite RO (2017). Prevalence and factors associated with childhood visual impairment in Ethiopia. Clin Ophthalmol Auckl NZ.

[19] Ellison EW (2013). Universal eye health: increasing access for the poorest. Community Eye Health.

[20] Potter A, Debrah O, Ashun J, Blanchet K. Eye health systems assessment (EHSA): Ghana country report march 2013. Ghana Country Report, Sightsavers, Accra, Ghana, 2013.

[21] World Health Organization, Geneva. A Conceptual Framework for Action on the Social Determinants of Health. 2010. Available from: https://www.who.int/publications-detail-redirect/9789241500852. Accessed 2 Feb 2021.

[22] Committee on Educating Health Professionals to Address the Social Determinants of Health, Board on Global Health, Institute of Medicine, National Academies of Sciences, Engineering, and Medicine. A Framework for Educating Health Professionals to Address the Social Determinants of Health. Vol. 3, A Framework for Educating Health Professionals to Address the Social Determinants of Health. Washington (DC): National Academies Press (US); 2016. Available from: https://www.ncbi.nlm.nih.gov/books/NBK395979/. Accessed 2 Feb 2021. 27854400

[23] Kavanagh A (2020). Disability and public health research in Australia. Aust N Z J Public Health.

[24] Mopari R, Garg B, Puliyel J, Varughese S (2019). Measuring disability in an urban slum community in India using the Washington group questionnaire. Disabil Health J.

[25] Mörchen M, Zambrano O, Páez A, Salgado P, Penniecook J, Brandt von Lindau A, et al. Disability-Disaggregated Data Collection: Hospital-Based Application of the Washington Group Questions in an Eye Hospital in Paraguay. Int J Environ Res Public Health. 2019;16(17):1–16. Available from: https://www.ncbi.nlm.nih.gov/pmc/articles/PMC6747208/. 10.3390/ijerph16173085 PMC6747208 31450663

[26] Sprunt B, Hoq M, Sharma U, Marella M (2019). Validating the UNICEF/Washington group child functioning module for Fijian schools to identify seeing, hearing and walking difficulties. Disabil Rehabil.

[27] Cappa C, Mont D, Loeb M, Misunas C, Madans J, Comic T, et al. The development and testing of a module on child functioning for identifying children with disabilities on surveys. III: Field testing. Disabil Health J. 2018;11(4):510–8. 10.1016/j.dhjo.2018.06.004. 10.1016/j.dhjo.2018.06.004 PMC6526372 30049638

[28] Mogle J, Hill NL, Bhargava S, Bell TR, Bhang I (2020). Memory complaints and depressive symptoms over time: a construct-level replication analysis. BMC Geriatr.

[29] Rius A, Artazcoz L, Guisasola L, Benach J (2014). Visual impairment and blindness in Spanish adults. Ophthalmology.

[30] Mactaggart I, Polack S, Murthy G, Kuper H (2018). A population-based survey of visual impairment and its correlates in Mahabubnagar district, Telangana state. India Ophthalmic Epidemiol.

[31] Ghana Statistical Service (2018). Multiple Indicator cluster survey (MICS2017/18), survey findings report.

[32] West BT, Sakshaug JW, Aurelien GAS (2016). How big of a problem is analytic error in secondary analyses of survey data?. PLoS One.

[33] Salvi SM, Akhtar S, Currie Z (2006). Ageing changes in the eye. Postgrad Med J.

[34] Robinson BE, Feng Y, Fonn D, Woods CA, Gordon KD, Gold D (2011). Risk factors for visual impairment- report from a population-based study (C.U.R.E.S.). Invest Ophthalmol Vis Sci.

[35] Srinivasan S, Swaminathan G, Kulothungan V, Raman R, Sharma T (2017). Prevalence and the risk factors for visual impairment in age-related macular degeneration. Eye.

[36] Robinson B, Feng Y, Woods CA, Fonn D, Gold D, Gordon K (2013). Prevalence of visual impairment and uncorrected refractive error – report from a Canadian urban population-based study. Ophthalmic Epidemiol.

[37] Ulldemolins AR, Lansingh VC, Valencia LG, Carter MJ, Eckert KA (2012). Social inequalities in blindness and visual impairment: a review of social determinants. Indian J Ophthalmol.

[38] Altindag D, Cannonier C, Mocan N (2011). The impact of education on health knowledge. Econ Educ Rev.

[39] Zajacova A, Lawrence EM (2018). The relationship between education and health: reducing disparities through a contextual approach. Annu Rev Public Health.

[40] Kovarski C, Faucher C, Orssaud C, Carlu C, Portalier S. Effect of Visual Impairments on Academic Performance. Vis Impact Inst. 2015; Available from: http://visionimpactinstitute.org/wp-content/uploads/2015/04/EFFECT-OF-VISUAL-IMPAIRMENTS-ON-ACADEMIC-PERFORMANCE-English.pdf. Accessed 3 Feb 2021.

[41] Zheng Y, Lamoureux EL, Chiang PPC, Rahman Anuar A, Wong TY (2014). Marital status and its relationship with the risk and pattern of visual impairment in a multi-ethnic Asian population. J Public Health.

[42] Tetteh J, Fordjour G, Ekem-Ferguson G, Yawson AO, Boima V, Entsuah-Mensah K (2020). Visual impairment and social isolation, depression and life satisfaction among older adults in Ghana: analysis of the WHO’s Study on global AGEing and adult health (SAGE) Wave 2. BMJ Open Ophthalmol.

